# Cholera outbreak in Syria amid humanitarian crisis: the epidemic threat, future health implications, and response strategy – a review

**DOI:** 10.3389/fpubh.2023.1161936

**Published:** 2023-06-20

**Authors:** Stanley Chinedu Eneh, Sofya Admad, Abubakar Nazir, Francisca Ogochukwu Onukansi, Alese Oluwatobi, David Chinaecherem Innocent, Temitope Olumuyiwa Ojo

**Affiliations:** ^1^Community Health Department, Obafemi Awolowo University, Ile-Ife, Osun State, Nigeria; ^2^Oli Health Magazine Organization (OHMO), Kigali, Rwanda; ^3^Centre for Infectious Diseases Research (CIDR), Nigeria Institute of Medical Research (NIMR) Yaba, Lagos, Nigeria; ^4^Medical Relief for Syria, Al-Hasakah, Syria; ^5^Sociology Department, Damascus University, Damascus, Syria; ^6^Department of Medicine, King Edward Medical University, Lahore, Pakistan; ^7^Department of Public Health, Federal University of Technology Owerri, Owerri, Imo State, Nigeria; ^8^Community Health Department, Obafemi Awolowo University Teaching Hospital Complex, Ile-Ife, Osun State, Nigeria

**Keywords:** cholera, outbreak, conflict, chlorination of water, war & Pollution, humanitarian crisis, Syria

## Abstract

The war in Syria, which started over 11 years ago, has devastated the country’s water sources, healthcare system, and other vital facilities for healthy living. The country is vulnerable to outbreaks, especially epidemic-prone ones like cholera, due to its fragile health system. Syria experienced its last hit of cholera in 2009, which led to the deaths of several Syrian children and affected about 1,000 people. The current cholera resurgence in Syria calls for public concern. Considering the poor access to clean water, the forced relocation of people, and other destruction caused by the war, these factors have exposed Syrian children to infectious diseases like cholera. We argued for more efforts toward the implementation of Water, Sanitation and Hygiene (WASH) in the country. We also pointed out the need for proper education and sensitization campaigns using all available resources to educate the populace, mass chlorination of wells, mapping vulnerable areas, and implementing WASH while encouraging vaccination coverage for cholera as a strategy to reduce its incidence. Improving the national surveillance systems will aid in the timely and appropriate reporting of any outbreak. Again, more negotiations should be done to seek a lasting solution to ending the war and restoring peace and serenity in the country.

## 1. Introduction

In many underdeveloped and low-income countries, cholera—an ancient disease—continues to be a global health challenge (1). Cholera infections affect both children and adults who consumed water and food contaminated with the bacterium *Vibrio cholerae* (2). Annually, over 2.8 million people are affected by cholera, resulting in more than 94,000 deaths globally (3). With the outbreak of the COVID-19 (Corona virus) pandemic, more harm and threats are posed to the global economy and healthcare system, resulting in an increase in emerging and re-emerging diseases. Currently, Syria has reported cholera outbreaks during its reoccurring civil wars (1).

There are an estimated 12 million people in need of drinking water and other WASH services in Syria due to the conflict, which has resulted in over 10,000 suspected cholera cases. Following this current situation, approximately 70% of the sewage is untreated, and nearly a quarter of the population relies on dangerous alternate water sources like water trucks, making cholera outbreaks in the country a constant threat (2). Cholera is a disease that reflects social protection levels, availability of clean water, sanitation, and hygiene, as well as population density (1, 2). Of the 6.5 million Syrians who are internally displaced, roughly 500,000 civilians—mostly women and children—are compelled to live in filthy, overcrowded settlements and communal shelters, where they stand at risk of contracting diseases like cholera and others that are spread by polluted water (2).

The outbreaks of cholera in Syria were previously reported in 2009, which led to the mortality of several Syrian children and about 1,000 other people (3). However, the conflict in Syria has also been reported to have affected the health system of the country, coupled with the huge impact of COVID-19, making early detection of disease outbreaks in Syria difficult.

On September 10, 2022, the Syrian Health Ministry confirmed a cholera outbreak in the Aleppo Governorate of Syria after 15 laboratory cases, one of which resulted in death (4). According to the data released between August 25 and September 10, 2022, there were 936 cases of severe acute watery diarrhea reported in Syria, including at least eight fatalities (4).

## 2. Syrian’s current health workforce and health budgeting

A shortage of human resources for health is more evident in certain areas of Syria. It was reported that some parts of Syria had one doctor per 800 people, while others had one doctor per 7,000 people since 2015 (5).

This conflict has also led to the mass destruction of health facilities, which reduces the services rendered by these facilities or even leads to their closure (6). The Syrian healthcare funding system is poor and limited. This shows a deep gap in knowledge around the country’s health care funding. According to the available data, domestic general government health expenditure as a percentage of Gross domestic product (GDP) stood at only 2% resulting to alarming out-of-pocket expenditures (7, 8).

## 3. The burden of COVID-19 in the pre-cholera outbreak in Syria

The first verified case of the COVID-19 pandemic in Syria was reported on March 22, 2020, and since then, the virus has spread widely throughout the nation (9, 10). However, the protracted conflict in Syria seriously damaged the health system and destroyed its structures. The Syrian health system lacks sufficient facilities and has outdated technologies, which makes the control and testing of the virus difficult (11). Furthermore, studies have shown that many trained physicians and more than 70% of the healthcare workforce fled the country due to conflict, making the country vulnerable to cholera outbreaks (12–15).

## 4. The epidemiology of cholera outbreak in Syria

Syria experienced frequent small-scale cholera epidemics in the first few decades following their independence, with approximately 11,000 illnesses in all governorates as of 1993, which was the deadliest the country had ever recorded (16). In 2008–2009, 390 incidents of cholera were reported in the north-eastern governorates of Deir al–Zour and Raqqa, but nothing more was heard of the illness for the following 13 years, until the war began (17). However, between February 1 and February 29, 2016, a water quality assessment was conducted in the northern city of Syria, and 61 drinking water samples were collected for chemical and biological analysis. The study indicated that 97% (59/61) and 80% (49/61) of the drinking water samples were biologically contaminated with *E. coli* and nitrates, respectively (18).

The cholera epidemic in Syria poses a serious risk to the area. Although the true numbers are likely under reported (4), According to the United Nations, the source of the outbreak is believed to be contaminated drinking water and agricultural irrigation from the Euphrates River. “This outbreak is also an indicator of severe shortages of water throughout Syria.” With the Euphrates level continuing to decrease, spiking drought-like conditions become inevitable, which leads to more competition for water by both man and animals and subsequent damages to the river bodies. Also, the extent of the destruction of the national water system makes the vulnerable population of Syria reliant on unsafe water sources, which may lead to the spread of dangerous waterborne diseases, particularly among children (4).

**Figure 1:**
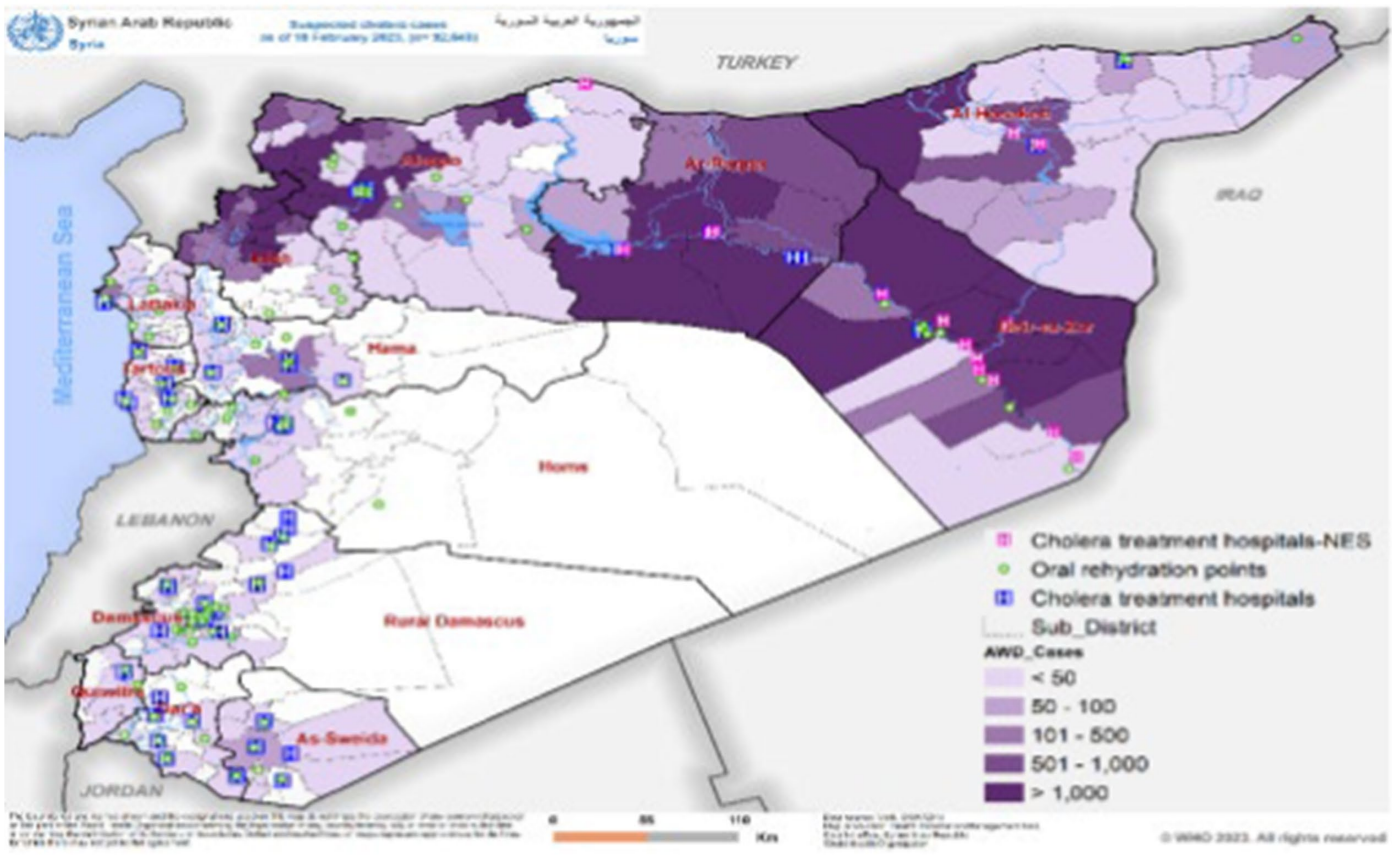
Showing the distribution of suspected and confirmed cholera cases as of 18 February, 2023 (Figure 1 was adopted from WHO Syrian Arab Republic: cholera outbreak situation report No. 13, issued 28 February, 2023).

As of February 28, 2023, the most affected governorates were Idleb, with 27,683 cases (30%), Aleppo, with 22,123 cases (23.9%), Deir ez-Zor, with 20,671 cases (22.3%), and Ar-Raqqa, with 17,578 cases (19%) (19, 20) (Figure 1).

**Figure 2:**
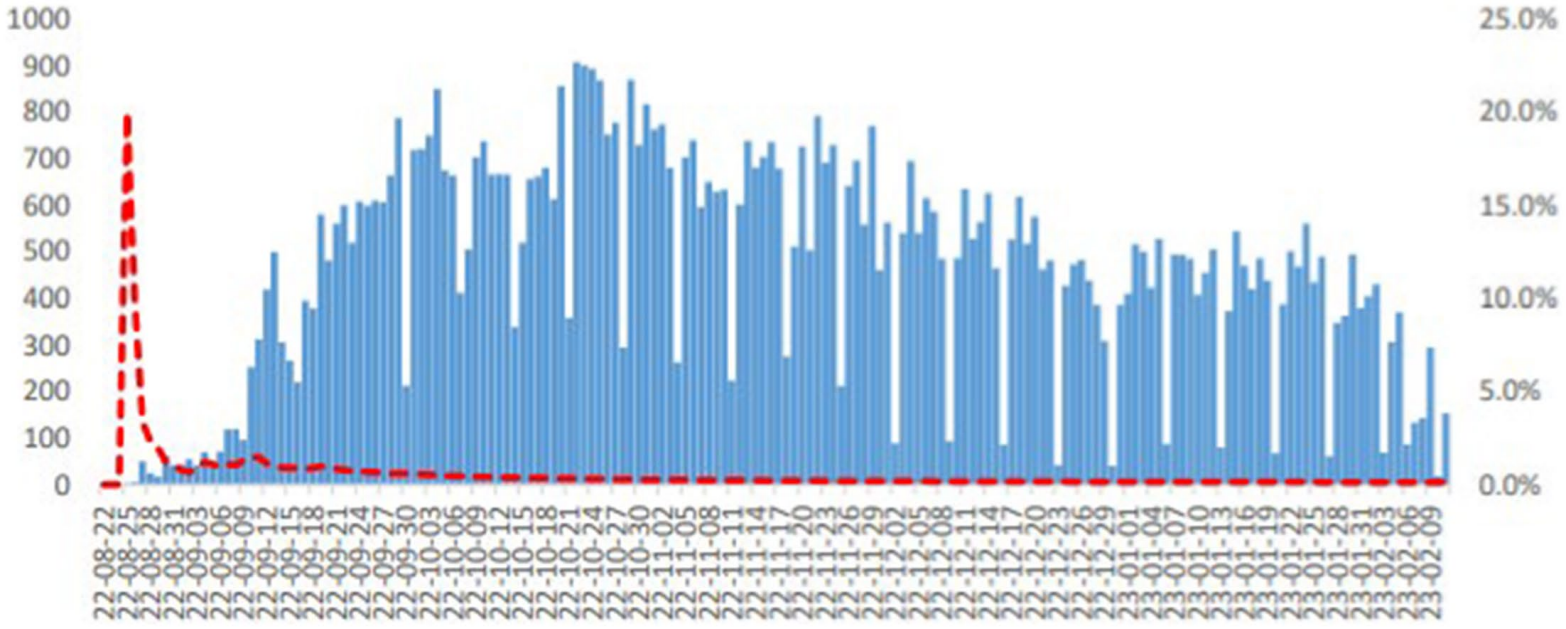
Showing the distribution of suspected cholera cases in Syria by date of onset, as of 23 February, 2023. (Figure 2 was adopted from WHO Syrian Arab Republic: cholera outbreak situation report No. 13, issued 28 February, 2023).

Between August 25, 2022 and 15 February, 2023, an estimated 92, 649 suspected cholera cases were reported in Syria’s fourteen governorates with a total of 101 cholera-related deaths during this time period, with Aleppo accounting for the majority 46 deaths, and Homs, Hama and Damascus recorded minority 1 death. The case fatality rate (CFR) is currently 0.11%, and the overall attack rate is 0.44% (19). 2,729 fecal specimens were studied and cultured, and 1,491 of those samples tested positive for *Vibrio cholerae*, serotype Ogawa EI Tor (1, 19) (Figure 2). And ever since the last Syrian’s cholera outbreak in 2009, no other serotype has been reported.

## 5. Current situations, challenges and causes of Syrian cholera outbreak

According to the International committee of red cross (ICRC), many thousands of Syrians still live in overcrowded shelters, and half the water supply chain is non-functional or damaged due to the conflict, which has raised concerns about the safety of drinking water. The damages to the PH level of the water, poor hygiene, a lack of water body treatment, and general Syrian environmental disasters could favor the wide spread of *V. cholerae* in the country (21). The amount of safe water available has been reduced to 5–30% of what it was before the crisis (22). The situation is worsened by the shortage of fuel because the working water plants cannot function without fuel, causing further exposure to sewage and unsafe drinking water.

The ongoing conflict in Syria has affected the country badly by damaging the infrastructure and impairing the healthcare services, sanitation systems, and availability of safe water and electricity, and has also had an impact on neighboring countries such as Lebanon and Iran. According to the United Nations (UN), more than two-thirds of water treatment plants in Syria have either been damaged or destroyed during the war since 2011 (23, 24). However, the current conflict, as well as ongoing military pressure on civilians, have resulted in the massive migration of millions of people into overcrowded, unsanitary, and filthy shelters with insufficient access to clean water. Engineers and maintenance crews are also criminalized for maintaining water plants in regions that are beyond the territory of government control (25).

The war has had so many effects on the entire population of Syria and also on its environment, one of which is the contamination of its water sources. The battle has destroyed almost two-thirds of the facilities used in the treatment of water: 50% of pumping stations, 1/3 of water towers, 25% of sewage treatment plants, and one-sixth of wells (1). About 47% of the Syrian population depends on alternate water sources to supplement or satisfy their needs for water, which is mostly unsafe due to the massive devastation of water bodies and sanitation due to the war (1). and the current earthquake in Syria had an overall impact on cholera responses (19).

## 6. Current effort to mitigate cholera outbreak in Syria

More than 1 million doses of cholera vaccine have been sent by the United Nations. On September 19, 2022, 30 tons of supplies were provided by the UN to the Syrian government to cope with the crisis (4). Aside from vaccines, water purification systems and medical assistance have been provided. Over 19 water treatment centers are currently installed. The World Health Organization (WHO) is aiming to strengthen the surveillance system in Syria to point out and track the cases, along with providing the appropriate treatment to the infected patients (25, 26).

The United Nations International Children’s emergency funds (UNICEF) is playing its role in close collaboration with WHO and targets to reach 690,000 people in the next three months for its WASH program, as well as cholera response planning, which will be carried out in all regions of Syria by UNICEF (27).

A reserve allocation of 25 million U.S. dollars has been launched by the Syria-cross-border humanitarian fund (SCHF) on September 19, 2022, with the aim of providing basic necessities such as strengthening WASH program and health interventions. The funding is also intended to improve emergency preparedness, particularly during the upcoming winter when conditions are expected to worsen (1, 25).

## 7. Future recommendations

Complications can be reduced by educating and training people on how to manage cholera at home, especially in mild cases, by using re-hydration fluids and clean home-based fluids. Resource-limited regions like rural areas require extra attention to limit the transmission of cholera. This can be achieved through the use of social media, short documentaries, and e-posters in the regional language for better understanding. These means should demonstrate the effectiveness of the WASH program and the use of current, pre-qualified, approved oral cholera vaccines (OCV) from the World Health Organization, such as Dukoral, Shanchol, and Euvichol-Plus, to lower the risk of cholera in Syria and other affected Asian and African countries (28).

However, intravenous and oral hydration have been confirmed to reduce the cholera-related mortality rate and remain treatments for cholera (29), which should be used in a moment like this. Antibiotics can be used as a hydration treatment for cholera, which has been confirmed to be effective for cholera treatment ever since 1964. In addition to this claim, a randomized controlled trial indicated that antibiotics such as tetracycline macrolides and fluoroquinolones can reduce diarrheal duration by 49–50%, the output and volume of stool by 8–92%, and the positive bacterial duration culture by 2–38% (30, 31).

Furthermore, usage of antibiotic regimes such as tetracycline, doxycycline (300 mg), which is equivalent to tetracycline, and active oral cholera vaccination (Dukoral, Shanchol, and Euvichol-Plus) (32) should be applied as a realistic and affordable preventive strategy in areas with conflicts and humanitarian crises, like Syria, during outbreak risk. Useful management tips should be spread via digital media. Research regarding cholera transmission should be encouraged, along with evaluating the effectiveness of various diagnostic tests. Further improved and enhanced vaccines should be developed to ensure long-term prevention (26).

WASH should be implemented to control and reduce the transmission of cholera (33). Awareness campaigns regarding the promotion of personal hygiene, the treatment and disinfection of household water, the chlorination of contaminated water, and the safe disposal of sewage should be conducted (34).

During the ongoing conflict in Syria, effective surveillance should be conducted according to World Health Organization recommendations. To this end, Syrian governments should grant NGOs operational independence. Laboratory diagnosis of cholera is not feasible amid the conflict in Syria, so the lack of prompt confirmation of cholera by laboratory methods should be supplemented by screening tests along with clinical diagnosis to decrease the rate of undetected cases.

On the other hand, antibiotic resistance is a serious global health concern. Considering the challenge of diagnosing cholera in the laboratory, rapid diagnostic tests (RDT) are advised with appropriate criteria to make the differential diagnosis of cholera and treat acute watery diarrhea (AWD) as well, and further research should be conducted to develop effective RDT for cholera (33).

## 8. Conclusion

War is a factor that makes a nation vulnerable to both human and natural attacks. The war in Syria has made the outbreak of cholera in the country unavoidable. Therefore, with the ongoing war destroying sources of clean water and health care systems, which in turn drastically reduces the systems’ strength to cater to the needs of citizens and manage disease outbreaks. War and other poor sanitation and hygiene practices play a triggering role in worsening or evoking disease outbreaks like cholera. The several conflicts of interest promulgated by government actions in reducing the reach of humanitarian aid and possible poor health funding instigate a double burden of crises in Syria. To control the current outbreak, the country must first achieve peace and unity, then implement WASH and increase vaccine coverage.

## Author contributions

SE: conceptualization, designing, project administration, investigation, writing – original draft, second draft, final draft, and writing-review and editing. SA: conceptualization, writing-original draft, collection and data assembly, second draft, and manuscript review and editing. AN: writing – original draft, collection and data assembly, second draft, and writing-review and editing. FO: writing – original draft, second draft, and writing-review and editing. AO: writing – original draft, writing-review and editing. DI: writing-original draft and writing – review & editing. TO: redesigning, project administration, final draft, Second draft, final writing-review and editing, and supervisor. All authors contributed to the article and approved the submitted version.

## Conflict of interest

The authors declare that the research was conducted in the absence of any commercial or financial relationships that could be construed as a potential conflict of interest.

## Publisher’s note

All claims expressed in this article are solely those of the authors and do not necessarily represent those of their affiliated organizations, or those of the publisher, the editors and the reviewers. Any product that may be evaluated in this article, or claim that may be made by its manufacturer, is not guaranteed or endorsed by the publisher.

## Abbreviations

SCHF: Syria-cross-border humanitarian fund
ICRC: International committee for red cross
WHO: World Health Organization
UNICEF: United Nations International Children’s emergency funds
UN: United Nations
WASH: Water, Sanitation and Hygiene
RDT: Rapid diagnostic tests
OCV: Oral cholera Vaccine
NGOs: None governmental organization
GDP: Gross Domestic Product
